# New vegetable varieties of *Brassica rapa* and *Brassica napus* with modified glucosinolate content obtained by mass selection approach

**DOI:** 10.3389/fnut.2023.1198121

**Published:** 2023-07-13

**Authors:** S. Coves, P. Soengas, P. Velasco, J. C. Fernández, M. E. Cartea

**Affiliations:** Group of Genetics, Breeding and Biochemistry of Brassicas, Misión Biológica de Galicia, Spanish Council for Scientific Research (MBG-CSIC), Pontevedra, Spain

**Keywords:** secondary metabolites, divergent selection, quality crops, landraces, plant defense

## Abstract

**Background:**

Glucosinolates (GSLs) constitute a characteristic group of secondary metabolites present in the *Brassica* genus. These compounds confer resistance to pests and diseases. Moreover, they show allelopathic and anticarcinogenic effects. All those effects are dependent on the chemical structure of the GSL. The modification of the content of specific GSLs would allow obtaining varieties with enhanced resistance and/or improved health benefits. Moreover, the attainment of varieties with the same genetic background but with divergent GSLs concentration will prompt the undertaking of studies on their biological effects.

**Objective and Methods:**

The objective of this study was to evaluate the efficacy of two divergent mass selection programs to modify GSL content in the leaves of two *Brassica* species: nabicol (*Brassica napus* L.), selected by glucobrassicanapin (GBN), and nabiza (*Brassica rapa* L.), selected by gluconapin (GNA) through several selection cycles using cromatographic analysis.

**Results:**

The response to selection fitted a linear regression model with no signs of variability depletion for GSL modification in either direction, but with higher efficiency in reducing the selected GSL than in the increasing. The selection was also effective in other parts of the plant, suggesting that there is a GSL translocation in the plant or a modification in their synthesis pathway that is not-organ specific. There was an indirect response of selection in other GSL; thus this information should be considered when designing breeding programs. Finally, populations obtained by selection have the same agronomic performance or even better than the original population.

**Conclusion:**

Therefore, mass selection seems to be a good method to modify the content of specific GSL in *Brassica* crops.

## Introduction

The *Brassica* genus belongs to the Brassicaceae family and is the most economically important genus within this family as *Brassica* crops represent the basis of world supplies along with cereals (1). The world production of *Brassica* genus vegetables has increased considerably throughout recent decades. Cultivated area has grown from 1.6 million ha to 2.4 million ha from 1990 to 2020; while production has increased from 39.3 million tons to 70.9 million tons during the same time frame (2). These crops exhibit an engaging nutritive profile since they provide considerably high amounts of fiber and protein when compared to other horticultural crops (3), besides having high antioxidant activity. This is related to phenolic compounds, especially flavonoids, and vitamin C contents (4–7) although it is also related to the carotenoid (8) and vitamin E (5) content. However, what differentiates *Brassica* crops from other horticultural crops is the presence of the secondary metabolites called glucosinolates (GSL).

GSL are a major class of secondary metabolites derived from amino acids. Their defining core structure is derived from select amino acids and comprises a β-thioglucosyl N-hydroxysulphate, containing a side chain and a β-glucopyranose moiety. GSL are grouped into three chemical classes based on the precursor amino acid: they are aromatic if derived from phenylalanine or tyrosine; aliphatic if derived from methionine, alanine, valine, leucine or isoleucine; and indolic if derived from tryptophan (9). Biosynthesis of GSL proceeds in three stages: (1) side-chain elongation of amino-acids, (2) development of the core structure, and (3) secondary side-chain modifications. Both extensive GSL side-chain modification and amino acid elongation are responsible for the more than 120 reported structures that show the chemical diversity of these compounds (10). When cellular damage occurs, GSL come into contact with plant myrosinase, a β-thioglucosidase that activates the generation of an unstable intermediate leading to the formation of biologically active chemicals, including nitriles, epithionitriles, thiocyanates, oxazolidine-2-thiones and/or isothiocyanates (11, 12).

GSL derived chemicals are recognized for both their role in plant defense and human health. After tissue breakdown caused by pests and necrotrophic pathogens, GSLs are hydrolyzed into toxic products. Toxicity is caused by changes in the permeability of cell membranes, the stability of DNA, the regulation of the cell cycle and mitochondrial function of plant pests and pathogens. Moreover, the system GSLs-myrosinase is involved in the stability of the cuticle from plant surfaces, the stomatal opening, and on the signaling in the plant’s innate immune response to microbial pathogens (12). Hydrolytic products from GSL have a chemo protective effect in humans. Two major groups of breakdown products, named isothiocyanates and indoles, have reported activity against many types of cancer in different stages of development by inducing detoxification enzymes (phase II) and inhibiting the activation of phase I enzymes. They also regulate cancer cell development by blocking the cell cycle and promoting apoptosis (13).

The increase in GSL content with health benefits and the reduction in the content of the harmful ones has been one of the main objectives in the improvement of *Brassica* crops. In this way, new varieties of rapeseed with no progoitrin and broccoli varieties with increased content of glucoraphanin have been produced. Progoitrin has a goitrogenic effect that has been proven in animals; whereas, glucoraphanin intake is related to the reduction of cancer risk or the maintenance of cardiovascular health (14). Different methods have been employed to modify the content of GSL in plants, such as genetic manipulation or crossbreeding (15–18). Obtaining new *Brassica* varieties with modified GSL profiles is interesting, not only to have new products with benefits in human nutrition, but also to obtain valuable starting material for genetic studies on the role of GSL in the defense against pests and/or pathogens.

Vegetable crops from the species *Brassica rapa L.* and *Brassica napus L.* are broadly cultivated and consumed in northwest of Spain. *B. napus* is cultivated for its leaves, receiving the local name of “nabicol” (*B. napus*+). *B. rapa* is also cultivated for its leaves, receiving the local name of “nabiza.” Crops of the both species are employed for human consumption in soups and stews during the winter period. At the end of the vegetative period and before flowering, tops of nabicol and nabiza are also employed for human consumption. Tops consist on fructiferous stems with flower buds and surrounding leaves, which are typically consumed while still green and before flower opening (19).

We carried out two mass selection programs to modify the leaf concentration of the GSL named glucobrassicanapin (GBN) in nabicol and gluconapin (GNA) in nabiza, to obtain new varieties with the same genetic background but different frequencies of those alleles that determine the character of interest.

Leaves of both crops are consumed as horticultural crops in northwestern Spain, hence the importance of making the selection in this particular plant organ for each specific GSL. Although previous studies have used mass selection as a method to modify GSL content in other *Brassica* species (17, 20), this is the first study that analyses the effects of mass selection in *B. napus* and *B. rapa*. Therefore, the main objective of this paper was to stablish the effectiveness of a divergent selection program to modify the content of GBN in *B. napus* and GNA in *B. rapa* leaves. Although *B. napus* and *B. rapa* are consumed mainly by their leaves, consumption of tops of the plant is also a common practice in northwest of Spain. Therefore, it is interesting to measure the indirect effect of selection in this part of the plant and in the seed that potentially can be employed to obtain oil of for fodder. Based on previous research, modification of a specific GSL have indirect effects in the concentration of other GSL of the plant and may also affect the agronomical performance of the crop. Therefore, a second objective was to measure indirect effects of the selection to modify GSL concentration (17).

## Materials and methods

### Divergent selection program evaluation and experimental design

Two landraces of northwestern Spain (Pontevedra) from *Brassica* genus kept at the Germplasm Bank at Misión Biológica de Galicia (MBG) were used in two independent and divergent selection programs. MBG-BRS0163 is a nabiza variety (*B. rapa*), and MBG-BRS0063 is a nabicol variety (*B. napus*). The divergent selection program in *B. rapa* started in 2008, while the one in *B. napus* started in 2011. The schedule followed in both species was similar. Approximately 250 plants of cycle 0 were transplanted into two different isolation cages. In each one of them, the leaf GSL content of all the plants was assessed 120 days after sowing by Ultra-High-Performance Liquid-Chromatograph (UHPLC). After analysis, 20% of the plants with the desired characteristic (high or low GSL content) were selected, and the rest of plants were removed before flowering. Cross-pollination among the selected plants in each cage was performed by bumblebees (*Bombus terrestris*). The selected plants were recombined with each other, and their seed mixture gave rise to the next cycle or generation. Following this schedule, six selection cycles were obtained in *B. rapa* and four in *B. napus* in both directions of selection for the target GSL, to increase (HGNA, HGBN) and decrease its content (LGNA, LGBN). To study the direct and indirect effects of selection, we have evaluated cycles C0, C1, C3, and C6 from *B. rapa* and cycles C0, C2, and C4 from *B. napus* (Table 1).

**Table 1:** List of selection cycles evaluated to test the effect of the divergent selection to modify the content of gluconapin (GNA) in *Brassica rapa* and glucobrassicanapin (GBN) in *Brassica napus*.

Species	Varieties^a^	Sowing date	Transplanting date
*Brassica rapa*	MBG-BRS0163 (C0)	01/09/2021	14/10/2021
	MBG-BRS0163 (HGNA) C1	01/09/2021	14/10/2021
	MBG-BRS0163 (HGNA) C3	01/09/2021	14/10/2021
	MBG-BRS0163 (HGNA) C6	01/09/2021	14/10/2021
	MBG-BRS0163 (LGNA) C1	01/09/2021	14/10/2021
	MBG-BRS0163 (LGNA) C3	01/09/2021	14/10/2021
	MBG-BRS0163 (LGNA) C6	01/09/2021	14/10/2021
*Brassica napus*	MBG-BRS0063 (C0)	01/09/2021	21/10/2021
	MBG-BRS0063 (HGBN) C2	01/09/2021	21/10/2021
	MBG-BRS0063 (HGBN) C4	01/09/2021	21/10/2021
	MBG-BRS0063 (LGBN) C2	01/09/2021	21/10/2021
	MBG-BRS0063 (LGBN) C4	01/09/2021	21/10/2021

a HGNA, high gluconapin content; LGNA, low gluconapin content; HGBN, high glucobrassicanapin content; LGBN, low glucobrassicanapin content.

Seeds from selection cycles were sowed first in seedbeds under greenhouse conditions. Approximately, 6 weeks afterwards, all varieties were transplanted into the experimental station of MBG placed at Pontevedra (Salcedo, NW Spain, 42° 24′N, 8° 38′W). Transplanting was carried out in October, in the field when the plants reached 5–6 leaves development (Table 1). Each experimental plot consisted of two rows spaced 0.6 m apart and plants on each row were spaced 0.8 m apart. Plots were arranged in a randomized block design with three repetitions. Each variety was randomly assigned on each repetition. Due to the internal variability that characterizes local varieties, C0 plants were planted in two different plots on each repetition to increase sample size and minimize experimental error due to such variability. Divergent selections of *B. rapa* and *B. napus* were evaluated independently in parallel trials.

In each plot, three bulks of 5 plants each were made with leaf and tops samples and collected at the optimal time of consumption. Leaves were taken 4 months after transplantation. Tops were taken before flowering time, between 4 and 6 months after transplanting. Samples were frozen *in situ* in liquid N_2_, immediately transferred to the laboratory and frozen at −80 ⁰C. All samples were freeze-dried (BETA 2–8 LD plus, Christ) for 72–96 h. Dried material was powdered by using an IKA – A10 (IKA – Werke GmbH& Co.KG) mill, so the fine powder was used for GSL extraction. Seeds from the same varieties of both species were also analyzed by using three replications of each. They were also powdered for GSL extraction.

### GSL extraction

The purification technique was used according to the Sephadex/sulphatase protocol, described by Kliebenstein (21) with some modifications. Ten mg (+/− 1 mg) of each lyophilized powder sample was weighed, and two replicates were placed in 1,2 mL tubes using racks of 96 tubes.

For the extraction of GSL, 400 μL of 100% (v/v) methanol preheated at 70 ⁰C, 10 μL of 0.3 M lead acetate, 120 μL of MiliQ water preheated at 70°C and 10 μL of glucotropaeolin (GTP) as internal standard were used. Samples were mixed with a shaker (Retsch MM30) for 130 min at 25 1/s and, subsequently, incubated in the dark on a rotatory shaker (Orbital Midi, Ovan) at 250 rpm for 1 h. Tissues and proteins were precipitated by centrifugation at 3700 rpm for 12 min to use the resulting supernatant for the chromatography analysis. A 96-well 45 μL pore multiscreen filter plates loaded with A-25 Sephadex resin were used with a Millipore multiscreen column loader. Three hundred μL of MiliQ water was added, and the mixture was allowed to equilibrate for 2 to 4 h. After drying the columns by vacuum pump (Millipore), 150 μL of supernatant was transferred to the 96-well plates, and the liquid was removed again by vacuum pump. This was repeated once more to reach 300 μL of vegetal extract. Columns were washed 4 times with 100 μL of 60% (v/v) methanol and another 4 times with 100 μL of MiliQ water using a vacuum pump between each wash.

To desulfate GSL in the samples, 10 μL of MiliQ water and 10 μL of sulfatase solution were added to each column. Plates were incubated overnight at room temperature (22). Desulfoglucosinolates were eluted by placing a 96-well plate under the column plate and aligning both plates to collect material using a vacuum pump. Columns were washed twice with 100 μL of 60% (v/v) ethanol and twice with 100 μL of MiliQ water, so that the resulting samples were kept at −20 ⁰C until their analysis by UHPLC (Ultra High – Performance Liquid Chromatography).

### GSL identification and quantification

To identify and quantify GSL, 3 μL of plant extract was used. Chromatographic analyses were carried out on an Acquity UPCL^®^H-Class coupled to a DAD (Diode Array Detector) (Waters, MA, United States). The UHPLC column used was an Acquity UPCL^®^BEH C_18_ (50 × 2.1 mm ID 130 Å; 1.7 μm particle size) (Waters, MA, United States). The oven temperature was set at 35°C and compounds were detected at 227 nm. GSL separation was carried out through the following method in 25% acetonitrile (A) in water (B) with a flow of 0.6 mL × min^−1^: conditions are initially set at 5% A (v/v) and then the gradient was increased to 0.63 min at 10% A (v/v), to 1.25 min at 30% A (v/v), to 2.08 min at 70% A (v/v), arriving to 100% A at 2.71 min and maintaining concentration until 3.54 min. At 3.75 min, the initial conditions were restored and maintained until 4.58 min. Total processing time of each sample was ≈5 min. Data obtained were recorded on a computer with the Empower 3 (Waters) software. The type and amount of each GSL was estimated based on the comparison of retention times with standards using GTP as internal standard, so that quantification was expressed in μmol/g DW.

### Agronomic parameters

Morphological and agronomic traits were recorded on each plot, throughout the maturation cycle of both species. Early vigor was taken 1 month after transplantation through a subjective visual scale from 1 (very poor) to 5 (excellent). For each species, time to flowering was recorded, and the number of tops was counted approximately every 4 days in order to obtain the date on which 50% sprouting was reached in each plot and thus calculate the precise time of collection of tops. At the end of the vegetative period, when plants are at the optimum time for harvesting (approximately 5 months after sowing), the number of leaves and height of 10 plants per plot were recorded for both *B. napus* and *B. rapa*. Thirty leaves of different plants from each plot were also collected to obtain the value of their fresh weight. In order to obtain an estimate of leaf moisture, 5 fresh leaves per plot were weighed (fresh weight) and left to dry for a week in an oven at ≈70°C. After this time, leaves were weighed again (DW) to obtain a humidity value for each plot. The procedure was also carried out on the tops to obtain their moisture content per plot. Once the flowering period was over, secondary tops per plant of 10 plants from each plot were counted. Tops from each plot were weighed together to obtain the average weight per top of the plants per plot.

### Statistical analysis

The results of GSL quantification were statistically analyzed through the SAS program (SAS, 2011). To evaluate the differences between selection cycles, an analysis of variance was carried out with selection cycles of each species to study both the content of individual GSL and the content of aliphatic, indolic and total GSL in the three parts of the plant studied (leaves, tops and seeds), using the PROC GLM of SAS. Cycles were considered fixed factors, and repetitions were random factors. The means of the selection cycles were compared using the Least Significant Difference test from Fisher (LSD, *p* ≤ 0.05). To analyze the selection response to GBN and GNA in the three organs, simple linear regression analyses were performed with the PROC REG of SAS (SAS, 2011), where GSL under selection was the dependent variable and selection cycles as the independent variable. Indirect selection response on other GSL was evaluated in leaves, tops and seeds, using the selected GSL (GBN on *B. napus* and GNA on *B. rapa*) as the independent variable and the other GSL (individual GSL and the sum of aliphatic, indolic and total GSL) as dependent variables.

## Results and discussion

### Specific GSL profile and content in two species

We found nine GSL in *B. napus*. Five of them were aliphatic: progoitrin (PRO), glucoalyssin (GAL), gluconapolipherin (GNL), gluconapin (GNA), and glucobrassicanapin (GBN). Three of them were indolic: glucobrassicin (GBS), methoxyglucobrassicin (MeOHGBS), and neoglucobrassicin (NeoGBS). Finally, we also found the aromatic gluconasturtin (GNT). The indolic hydroxyglucobrassicin (OHGBS) was only found in *B. napus* seeds. *B. rapa* showed eight GSL. Four were aliphatic: PRO, GNA, OHGBS, and GBN; three indolic: GBS, MeOHGBS, and NeoGBS; and one aromatic: GNT. In *B. rapa* seeds, we also found the aliphatic gluconapoleipherin (GNL; Table 2).

**Table 2:** Classification of the GSL present in the analyzed samples of *Brassica. napus* and *Brassica rapa*.

Type	Chemical name	Common name	Abbr.	Species	Organ
Aliphatics	2-hydroxy-3-butenyl	Progoitrin	PRO	*B. napus*	L, T, S
				*B. rapa*	L, T, S
	5-methylsulpinlypentyl	Glucoalyssin	GAL	*B. napus*	L, T, S
	2-hydroxy-4-pentenyl	Gluconapolipherin	GNL	*B. napus*	L, T, S
				*B. rapa*	S
	3-butenyl	Gluconapin	GNA	*B. napus*	L, T, S
				*B. rapa*	L, T, S
	4-pentenil	Glucobrassicanapin	GBN	*B. napus*	L, T, S
				*B. rapa*	L, T, S
Indolics	3-indolylmethyl	Glucobrassicin	GBS	*B. napus*	L, T, S
				*B. rapa*	L, T, S
	4-hydroxy-3-indolylmethyl	Hydroxyglucobrassicin	OHGBS	*B. napus*	S
				*B. rapa*	L, T, S
	4-methoxy-3-indolylmethyl	Methoxyglucobrassicin	MeOHGBS	*B. napus*	L, T, S
				*B. rapa*	L, T, S
	1-methoxy-3-indolylmethyl	Neoglucobrassicin	NeoGBS	*B. napus*	L, T, S
				*B. rapa*	L, T, S
Aromatics	2-phenethyl	Gluconasturtiin	GNT	*B. napus*	L, T, S
				*B. rapa*	L, T, S

L, leaves; T, tops; S, seeds.

The concentration of total GSL at cycle 0 was higher in seeds, followed by tops and leaves in *B. napus*; whereas, in *B. rapa*, the highest GSL content was found in the tops, followed by seeds and leaves (Supplementary Table S1). The higher concentration of GSL in seeds and tops compared to leaves coincides with previous reports in *B. napus* (23–25). Accumulation of GSL in different organs of the plant is genetically regulated. For example, gene *BnaC02.GTR2* is a positive regulator of GSL accumulation in seeds but has a negative impact on vegetative tissue (26). Probably, when the plant is in the vegetative stage, it synthetizes GSL, which are later mobilized first to the flowers, and afterwards to the seeds. In this way, seeds have a high concentration of these secondary metabolites needed for defense in the first stages of germination, prior to being able to synthetize GSL by themselves.

Regarding the chemical class, aliphatic GSL predominated in all tissues of both species. They were in higher percentages in *B. rapa* than in *B. napus* (Supplementary Tables S1, S2). Indole GSL were more abundant in *B. napus* than in *B. rapa*, and, in both cases, they predominated in leaves, followed by tops and seeds. GNT was the only aromatic GSL detected, and it was found in higher proportion in all *B. napus* parts analyzed than in those of *B. rapa*. Both species showed tops as the part of the plant with higher GNT content, followed by leaves and seeds.

The profile of *B. napus* leaves was dominated by GBN, PRO, and GNA, in agreement with previous reports in the same species (27–30). However, the profile changed in other parts of the plant. In tops and seeds, the predominant GSL was PRO, followed by GBN and GNA in tops and GNA and GBN in seeds (Supplementary Table S1). GNA was the main GSL in the three organs of *B. rapa*, followed by GBN and MeOHGBS in leaves, GBN and GNT in tops and OHGBS and GBN in seeds. The profile of *B. rapa* agrees with that found by Kim (31), Padilla (32) and Francisco (19), who reported similar GSL `proportions to our results. Therefore, the profile of GSL in leaves is very similar to that found by other authors; however, the total concentration and profile changes in other parts of the plant agreed with previous reports in *Brassica* species, such as *B. oleracea* (17, 24, 29, 33, 34) or *B. napus* (23, 33).

### Direct response to divergent mass selection

**Figure 1:**
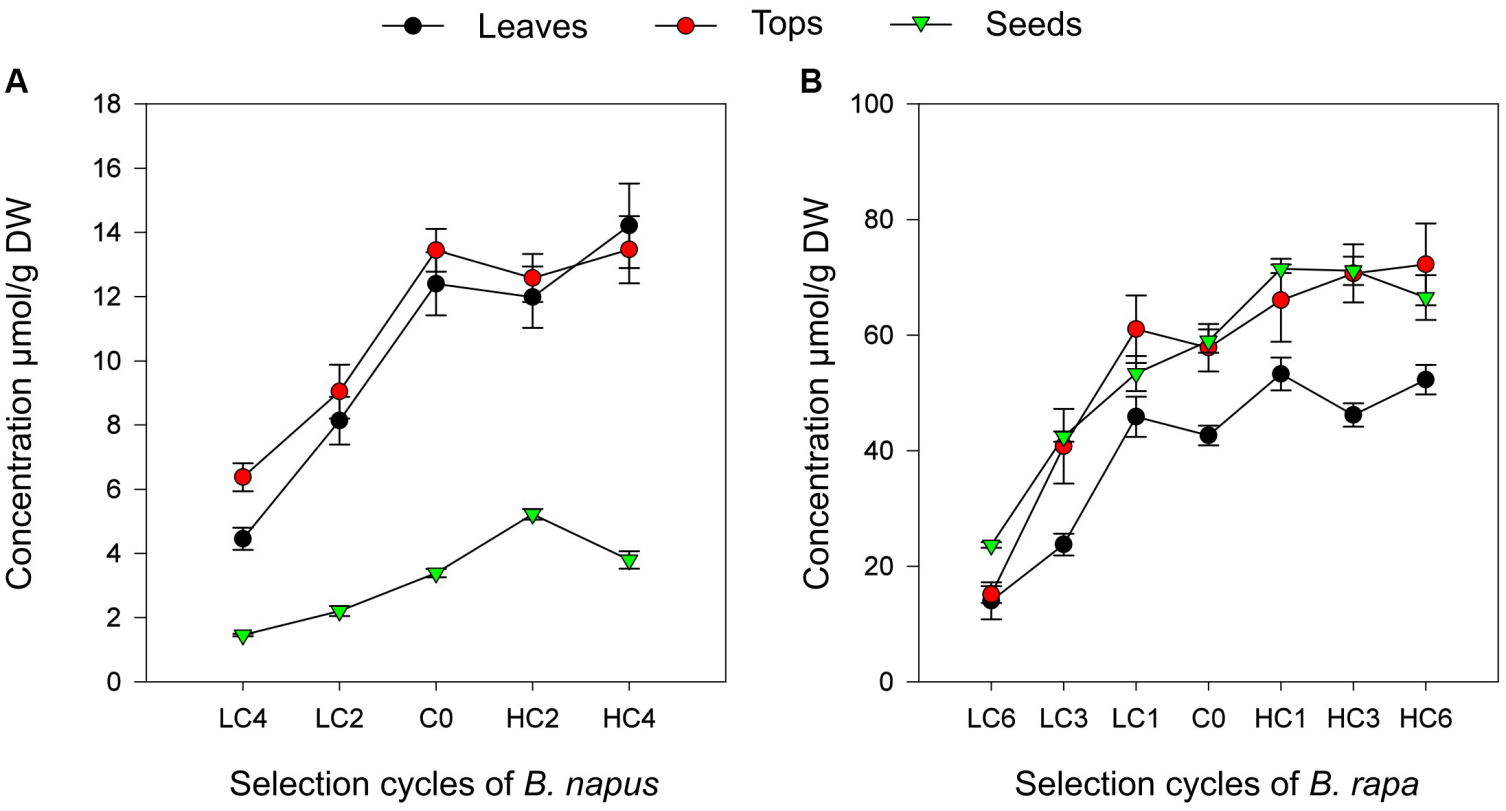
Linear regressions of GNA on selection cycles in *Brassica napus* **(A)** and GBN on selection cycles in *Brassica rapa* **(B)** in the three parts of the plant studied.

The regression coefficient of target GSL on selection cycles were positive and significant in leaves for GBN in *B. napus* (*R*^2^ = 0.852; *a* = 1.16839; *p* = 0.0162; Figure 1) and GNA in *B. rapa* (*R*^2^ = 0.6979; *a* = 3.30873; *p* = 0.0119; Figure 1). Therefore, it can be concluded that mass selection is an effective method to increase or decrease the concentration of individual GSL in these species. Moreover, our results suggest that the concentration of these compounds is a trait with a high heritability coefficient, although other authors have found that there is also a substantial contribution of non-additive gene effects (35). This study coincides with previous works where the efficacy of this method to modify the concentration of other GSL was tested in *B. rapa* (20) and *B. oleracea* (17).

However, the selection response was asymmetric, being more effective to decrease the content of the target GSL than to increase it, which agreed with Sotelo (17) and Korkovelos and Goulas (36). One of the possible explanations of this effect is the depletion in the variability to increase the GSL concentration. Evolutionarily, as a defensive mechanism (37–39), GSL accumulation may have provided certain advantages over plants with reduced GSL content (40). Possibly, through generations, a trend to increase them has occurred leaving a greater margin of genetic variability for GSL reduction.

Although selection was carried out in leaves in this work, we also analyzed the indirect response in other parts of the plant. The results showed a positive significant linear regression coefficient in GBN concentrations through selection cycles in *B. napus* tops (*R*^2^ = 0.7195; *p* = 0.0439). However, the regression of GBN in seeds was non-significant (Table 3; Figure 1). In *B. rapa*, positive significant linear regression coefficients were obtained for GNA content through cycles in tops (*R*^2^ = 0.7976; *p* = 0.0042) and seeds (*R*^2^ = 0.7346; *p* = 0.0085; Table 4; Figure 1). Therefore, modification of the concentration of the target GSL in leaves leads to modification in other plant organs. As it was observed in leaves, the response in other parts of the plant was also asymmetric. This indirect response may be due to a selection of genes operating in different parts of the plant or to a transport between them (15, 16).

**Table 3:** Simple linear regression coefficients of GSL concentration in *Brassica napus*.

	Leaves		Tops		Seeds	
	*R* ^2^	*a*	*R* ^2^	*a*	*R* ^2^	*a*
TOTAL	0.5686	1.04409	−0.2100	0.28783	0.1217	1.26128
Aliphatic	0.8560	1.51752^*^	0.5100	0.87265	0.1105	1.19871
PRO	0.3247	0.23338	−0.3321	0.01397	0.0874	0.55886
GAL	0.8684	0.18466^*^	0.6733	0.08546	0.7516	0.30173^*^
GNL	0.4596	−0.15655	0.3852	−0.12051	−0.2234	0.01015
GNA	0.5128	0.25604	0.2283	0.11039	−0.3160	0.06601
OHGBS	–	–	–	–	−0.2623	0.03205
GBN	–	–	0.9351	0.78334^**^	0.3318	0.26196
Indole	0.5922	−0.41990	0.4102	−0.40082	−0.2780	0.02962
GBS	0.6280	−0.37961	0.5256	−0.37042	−0.3333	0.000026
MeOHGBS	−0.2096	−0.00642	−0.3321	0.00084	−0.3187	−0.00345
NeoGBS	0.5032	−0.03386	−0.0206	−0.03124	−0.0749	0.00099
Aromatic						
GNT	0.5707	−0.05354	0.6721	−0.18401	−0.2178	0.03295

Leaf GBN content is used as the independent variable and the rest of GSL on leaves, tops and seeds are used as dependent variables. *R*^2^, determination coefficient for each GSL; a, slope. ^*^Significance at *p* ≤ 0.05 and ^**^significance a *p* ≤ 0.01.

**Table 4:** Simple linear regression coefficients on GSL concentration in *Brassica rapa*.

	Leaves		Tops		Seeds	
	*R* ^2^	*a*	*R* ^2^	*a*	*R* ^2^	*a*
TOTAL	0.9945	1.04433^**^	0.9103	1.40089^**^	0.8702	1.16193^**^
Aliphatic	0.9960	1.05808^**^	0.9059	1.37936^**^	0.8745	1.12653^**^
PRO	0.0012	0.00180	−0.1193	0.00130	0.1671	0.01507
GAL	–	–	–	–	–	–
GNL	–	–	–	–	0.7244	−0.00083^**^
GNA	–	–	0.9105	1.31225^**^	0.8723	1.10011^**^
OHGBS	0.8199	−0.01769^**^	0.8004	−0.01023^**^	0.5193	0.02970^*^
GBN	0.3822	0.05628	0.4842	0.06581^*^	0.5048	0.01218^*^
Indole	0.8466	−0.03570^**^	0.6547	−0.03336^*^	0.4437	0.03264
GBS	0.7564	−0.01048^**^	0.3349	−0.00771	−0.0278	0.00277
MeOHGBS	−0.0171	−0.00323	0.2703	−0.00901	−0.1503	0.00049
NeoGBS	0.4481	−0.00431	0.3100	−0.00641	−0.0375	−0.0003288
Aromatic						
GNT	0.3170	0.02196	0.6924	0.05489^*^	0.0460	0.00275

Leaf GNA content is used as the independent variable and the rest of GSL on leaves, tops and seeds are used as dependent variables. *R*^2^, determination coefficient for each GSL; a, slope. ^*^Significance at *p* ≤ 0.05 and ^**^significance at *p* ≤ 0.01.

### Indirect response to divergent mass selection in other GSL

**Figure 2:**
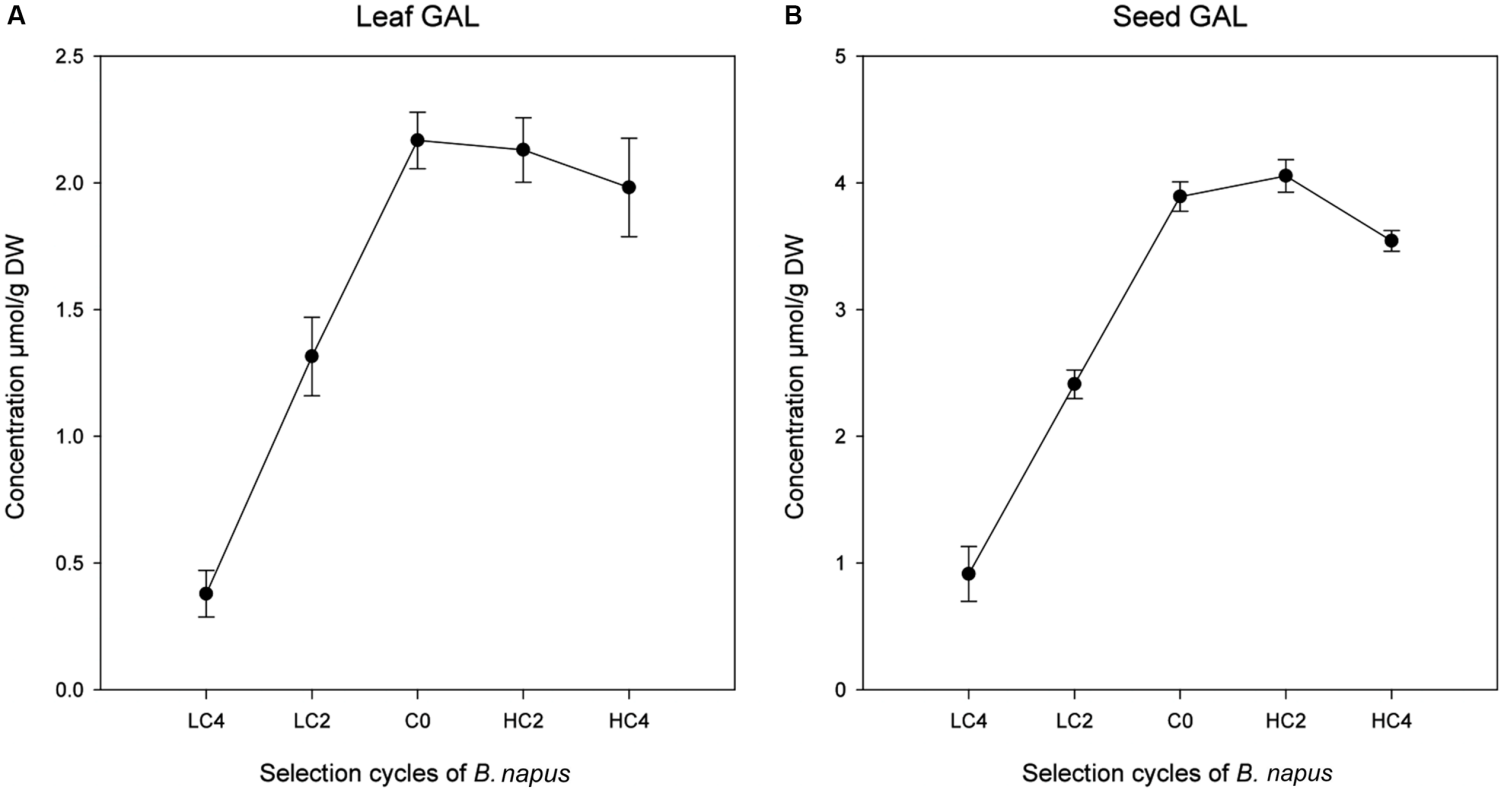
Linear regressions of other GSL with an indirect response to GBN selection cycles in *Brassica napus* in leaves **(A)** and seeds **(B)**.

A regression analysis using leaf GBN content as the independent variable and the rest of GSL as dependent variables was performed in the three parts of the plant analyzed (Table 3). We found significant positive regression coefficients of GBN with GAL in leaves (*p* = 0.0136) and seeds (*p* = 0.0326; Table 3; Figure 2). No significant regressions were found in tops. GAL is the precursor of GBN in the biosynthetic pathways of aliphatic GSL; therefore, we probably are selecting by one or several genes which are located before the step of GAL synthesis.

**Figure 3:**
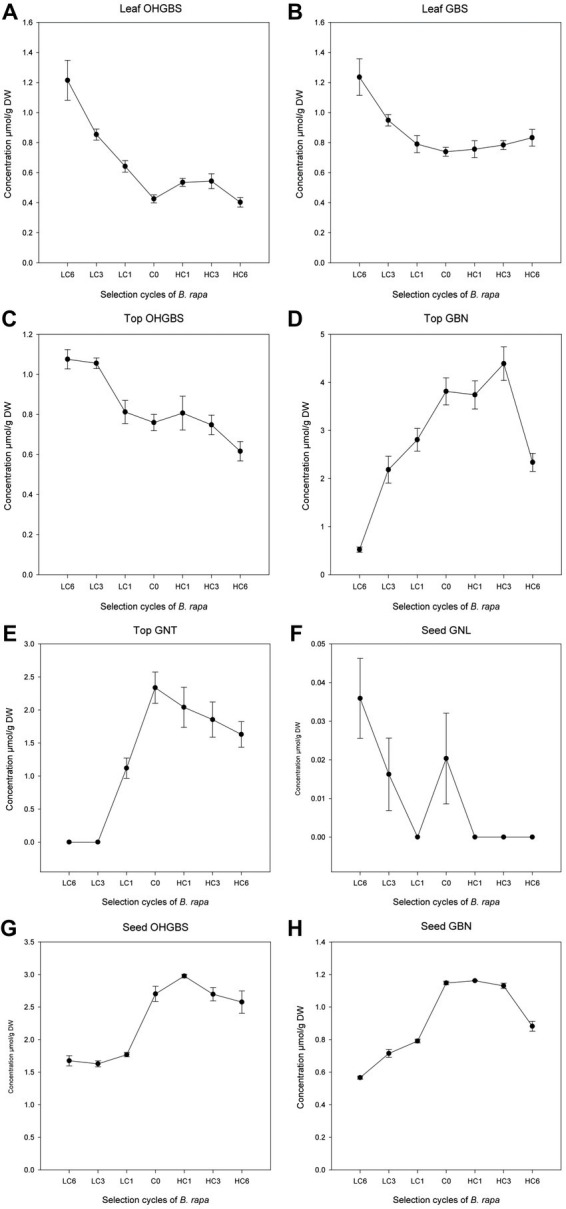
Linear regressions of other GSL with an indirect response to GNA selection cycles in *Brassica rapa* in leaves **(A,B)**, tops **(C–E)** and seeds **(F–H)**.

In the *B. rapa* selection, we found positive and significant regression coefficients of GNA with aliphatic and total GSL (Table 4; Figure 3) in the three parts of the plant analyzed. Negative regression coefficients with GBS (*p* = 0.0068), OHGBS (*p* = 0.0031), and indolic GSL (*p* = 0.0021) were found in leaves and with OHGBS (*p* = 0.0041) and indolics (*p* = 0.017) in tops. Therefore, when the concentration of GNA was modified, the concentration of aliphatic GSL and total GSL changed in the same direction as GNA; whereas, indolic GSL were modified in the opposite direction. These results make sense considering that GNA is aliphatic and the major GSL in this species. Aliphatic and indolic GSL are synthesized following two different pathways with independent regulation. The indirect effect on indolic GSL may respond to a cross-talk between both synthetic pathways (41) and to the need to save resources in defense. The concentration of other individual GSL was also modified in GNA divergent selection. In tops, we found positive and significant regression coefficients in GBN (*p* = 0.0497) and GNT (*p* = 0.0125), while in seeds, we found a positive indirect response in the concentration of OHGBS (*p* = 0.0410) and GBN (*p* = 0.0445). In seeds, we found a negative regression coefficient with GNL (*p* = 0.0094; Table 4; Figure 3). The indirect response of selection in other GSL has important implications in plant breeding. If we want to improve the concentration of a beneficial GSL, we may have undesired effects in other GSL, thus this information should be considered when designing breeding programs.

### Indirect response to divergent mass selection in agronomic parameters

The *B. napus* and *B. rapa* local varieties employed to start the divergent selection cycles showed in previous evaluations good agronomic performance to be cultivated as crops (13, 42, 43), as well as intra-variability for GSL content. Besides having a role in plant defense, GSL can also interfere with other processes in the plant. Some evidence suggests that there is cross-talk of the biosynthetic GSL pathway with the hormone metabolism, stomatal aperture, biomass, and flowering (44–47). Therefore, the modification of GSL concentration in plants may have indirect effects in morphological and agronomical characters in the cultivated varieties, which may impact the final production of crops or other traits related to its quality. In previous field evaluations, some authors have found negative correlations between the GSL content and the leaf colour in *B. juncea* (48), showing that the lighter the leaf colour, the higher the GSL contents. In the same species, Merah (49) found that both the total GSL and GNA content were positively related to seed yield, measured as seed filling duration and thousand seed weight. However, relationship between GSL content and agronomical and/or production traits has not been studied in other *Brassica* crops. In our experiment, an increment in the top number per plant (*R*^2^ = 0.4952; *a* = 0.04730; *p* = 0.0469) and a significant reduction in the top moisture (*R*^2^ = 0.5778; *a* = −0.08632; *p* = 0.0289) were related to an increase of leaf GNA content in *B. rapa*. Therefore, in *B. rapa* selection, population with increased GNA content have also a high number of tops, and low PRO content, thus they are interesting from an agronomical and human health point of view. We did not find any indirect effect of selection in agronomic parameters in the *B. napus* selection. As conclusion, populations obtained by mass selection have the same agronomic performance or even better than the original populations.

## Conclusion

Divergent mass selection was an effective method to quantitatively modify the leaf content of GBN in *B. napus* and GNA in *B. rapa*, suggesting that there is high genetic variability and heritability for GSLs content within the studied species. An asymmetrical response to selection was observed in both species as it was more effective to decrease selected GSL than to increase it. The selection was also effective in other parts of the plant, suggesting that there is a GSL translocation in the plant or a modification in their synthesis pathway that is non-organ specific. Both divergent selection programs on aliphatic GSLs had an indirect response on other aliphatic GSL, but only *B. rapa* selection showed indolic GSL response, mostly negative. The indirect response of selection in other GSL has important implications in plant breeding. Selection to increase the content of a specific GSL, may have undesired effects in other GSL, thus this information should be considered when designing breeding programs. Finally, populations obtained by selection have the same agronomic performance or even better than the original population. Therefore, mass selection seems to be a good method to modify the content of specific GSL in *Brassica* crops. The populations obtained in this study represent valuable materials to broaden understanding of the biological effects of GSL in these species. Also, we have obtained new *Brassica* varieties enriched in GSL content and with good agronomic performance that can be released into the market.

## Data availability statement

The original contributions presented in the study are included in the article/Supplementary material, further inquiries can be directed to the corresponding author.

## Author contributions

MC and PV conceived the project and designed the experiments. PS assisted with the setup of the plant experiments and supervised the manuscript. SC carried out the experiments and wrote the manuscript. MC and PS coordinated the work. JF assisted with the field work. All authors contributed to the article and approved the submitted version.

## Funding

This research was financed by the projects RTI2018-096591-B-I00 and PID2021-126472OB-I0 from the Ministry of Science and Innovation of Spain.

## Conflict of interest

The authors declare that the research was conducted in the absence of any commercial or financial relationships that could be construed as a potential conflict of interest.

## Publisher’s note

All claims expressed in this article are solely those of the authors and do not necessarily represent those of their affiliated organizations, or those of the publisher, the editors and the reviewers. Any product that may be evaluated in this article, or claim that may be made by its manufacturer, is not guaranteed or endorsed by the publisher.

## Supplementary material

The Supplementary material for this article can be found online at: https://www.frontiersin.org/articles/10.3389/fnut.2023.1198121/full#supplementary-material
