# Supplementary material for: New vegetable varieties of Brassica rapa and Brassica napus with modified glucosinolate content obtained by mass selection approach
Source: Front Nutr. 2023 Jul 13;10:1198121. doi: 10.3389/fnut.2023.1198121 (PMC10373736; doi:10.3389/fnut.2023.1198121)
Supplement: Supplementary file 1 [file Data_Sheet_1.PDF]

Table S1.- Individual and total GSL concentration (µmol/g DW) on each *B. napus* selection cycle. [1]Divergent selection studied GSL. Means of the same row with the same letter do not present signifficant diferences between them.

GSL	Leaves					
	**Bn63 (LGBN) C4**	**Bn63 (LGBN) C2**	**Bn63-C0**	**Bn63 (HGBN) C2**	**Bn63 (HGBN) C4**	**LSD**
**TOTAL**	30.7465 b	39.5913 ab	43.6140 a	37.2897 ab	41.6759 a	10.642
**Aliphatic**	21.8625 b	32.3119 a	36.5199 a	33.2140 a	37.8682 a	8.397
PRO	6.5376 b	9.2753 a	9.7212 a	7.9515 ab	9.2374 a	2.2328
GAL	0.3783 c	1.3155 b	2.1682 a	2.1307 a	1.9820 ab	0.7093
GNL	5.1667 ab	5.7875 a	3.8778 c	4.2641 bc	4.1756 bc	1.2105
GNA	5.3288 b	7.8030 a	8.3513 a	6.8836 ab	8.2650 a	1.9801
GBN[1]	4.4511 c	8.1306 b	12.4014 a	11.9842 a	14.2082 a	2.9959
**Indole**	7.1017 a	5.4506 a	5.5553 a	2.8169 b	2.4652 b	2.6073
GBS	5.9574 a	4.5349 a	4.4356 a	2.0769 b	1.8734 b	2.1955
MeOHGBS	0.5041 ab	0.4668 ab	0.5891 a	0.4044 ab	0.3826 b	0.2028
NeoGBS	0.6402 a	0.4489 abc	0.5306 ab	0.3357 bc	0.2092 c	0.2581
**Aromatic**						
GNT	1.7823 a	1.8288 a	1.5388 ab	1.2588 b	1.3425 b	0.3979

GSL	Tops					
	**Bn63 (LGBN) C4**	**Bn63 (LGBN) C2**	**Bn63-C0**	**Bn63 (HGBN) C2**	**Bn63 (HGBN) C4**	**LSD**
**TOTAL**	46.8765 ab	51.2639 ab	54.6436 a	46.0337 b	46.7592 ab	8.4261
**Aliphatic**	35.8162 b	43.1501 a	47.8170 a	41.3635 ab	42.8032 a	6.7631
PRO	13.6294 b	16.8212 a	16.3310 a	12.9509 b	13.5718 b	2.0871
GAL	1.8496 b	2.2460 ab	2.8882 a	2.5766 ab	2.3548 ab	0.9446
GNL	5.4984 a	5.4004 a	4.9687 ab	4.3787 bc	4.0118 c	0.9215
GNA	8.4661 b	9.6394 ab	10.1866 a	8.8765 ab	9.3900 ab	1.5778
GBN[1]	6.3726 c	9.0431 b	13.4426 a	12.5807 a	13.4659 a	2.3387
**Indole**	7.4804 a	4.5303 b	4.3824 b	2.6072 bc	2.1183 c	2.1736
GBS	6.2376 a	3.8972 b	3.3032 bc	2.0622 c	1.6552 c	1.7511
MeOHGBS	0.3196 ab	0.09947 b	0.3411 a	0.1859 ab	0.1341 ab	0.2236
NeoGBS	0.9231 a	0.5336 bc	0.7381 ab	0.3591 c	0.3289 c	0.3112
**Aromatic**						
GNT	3.5799 a	3.5836 a	2.4441 b	2.0630 bc	1.8377 c	0.5477

GSL	Seeds					
	**Bn63 (LGBN) C4**	**Bn63 (LGBN) C2**	**Bn63-C0**	**Bn63 (HGBN) C2**	**Bn63 (HGBN) C4**	**LSD**
**TOTAL**	56.8758 bc	54.2159 c	64.5974 b	76.0214 a	63.8980 b	9.088
**Aliphatic**	53.0959 bc	52.0142 c	60.7220 b	72.4276 a	60.4546 bc	8.535
PRO	33.0316 b	32.7838 b	37.6390 ab	42.2323 a	35.2563 b	5.312
GAL	0.9130 c	2.4119 b	3.8930 a	4.0558 a	3.5427 a	0.7542
GNL	0.7288 bc	0.6023 c	0.7457 b	0.9821 a	0.7167 bc	0.1424
GNA	16.9627 b	14.0088 c	15.0553 bc	19.9369 a	17.1447 b	2.2601
OHGBS	3.1786 a	1.8233 b	2.9747 a	2.5055 ab	2.9806 a	0.6925
GBN[1]	1.4597 d	2.2073 c	3.3891 b	5.2206 a	3.7942 b	0.5352
**Indole**	3.4865 a	2.2017 c	3.2310 ab	2.5758 bc	3.4434 a	0.7583
GBS	0.1183 a	0.08259 bc	0.09725 b	0.070735 c	0.1261 a	0.0204
MeOHGBS	0.1770 ab	0.2959 a	0.1402 ab	0 a	0.3211 a	0.2369
NeoGBS	0.01259 a	0 a	0.01889 a	0 a	0.01565 a	0.025
**Aromatic**						
GNT	0.2935 bc	0 c	0.6443 ab	1.0180 a	0 c	0.615

Table S2.- Individual and total GSL concentration (µmol/g DW) on each *B. rapa* selection cycle and original cycle. [1]Divergent selection studied GSL. Means of the same row with the same letter do not present signifficant diferences between them.

GSL	Leaves							
	**Br163 (LGBN) C6**	**Br163 (LGBN) C3**	**Br163 (LGBN) C1**	**Br163-C0**	**Br163 (HGBN) C1**	**Br163 (HGBN) C3**	**Br163 (HGBN) C6**	**LSD**
**TOTAL**	18.3837 d	28.3944 c	51.0032 b	50.3571 b	60.1723 a	52.0119 ab	57.1460 ab	8.3771
**Aliphatic**	14.5985 d	25.2452 c	48.3154 b	46.8753 b	56.9414 a	48.9201 b	54.0794 ab	7.9137
PRO	0.01811 ab	0 b	0.0689 b	0.1709 a	0.1251 ab	0.01527 b	0.0139 b	0.1388
GAL	-	-	-	-	-	-	-	-
GNL	-	-	-	-	-	-	-	-
GNA[1]	14.0176 e	23.7344 d	45.8538 bc	42.6139 c	53.2953 a	46.1643 abc	52.2963 ab	7.2499
OHGBS	1.2149 a	0.8535 b	0.6420 bc	0.4255 c	0.5357 c	0.5434 c	0.4029 c	0.2754
GBN	0.5629 e	1.5108 de	2.3928 cd	4.0904 a	3.5210 ab	2.7406 bc	1.7692 cd	1.0005
**Indole**	3.7851 a	3.1492 ab	2.6352 bc	2.2390 c	2.4147 c	2.5799 bc	2.1741 c	0.7065
GBS	1.2362 a	0.9489 b	0.7901 b	0.7395 b	0.7565 b	0.7841 b	0.8328 b	0.2851
MeOHGBS	0.9710 a	0.9031 ab	0.9040 ab	0.6965 b	0.8536 ab	1.0396 a	0.7270 b	0.2352
NeoGBS	0.3631 ab	0.4436 a	0.2991 bc	0.3775 ab	0.2689 bc	0.2129 c	0.2114 c	0.1367
**Aromatic**								
GNT	0 c	0 c	0.05256 c	1.2428 a	0.8162 ab	0.5118 bc	0.8925 ab	0.5187

GSL	Tops							
	**Br163 (LGBN) C6**	**Br163 (LGBN) C3**	**Br163 (LGBN) C1**	**Br163-C0**	**Br163 (HGBN) C1**	**Br163 (HGBN) C3**	**Br163 (HGBN) C6**	**LSD**
**TOTAL**	18.7774 c	46.3824 b	67.4734 a	66.4379 a	74.3150 a	78.7995 a	78.1719 a	17.332
**Aliphatic**	15.6339 c	42.9497 b	63.9992 a	61.8573 a	69.8182 a	75.0899 a	74.6022 a	16.721
PRO	0.009878 a	0.005610 a	0.1480 a	0.1835 a	0.03279 a	0.009850 a	0.01171 a	0.1842
GAL	-	-	-	-	-	-	-	-
GNL	-	-	-	-	-	-	-	-
GNA[1]	15.1022 c	40.7601 b	61.0442 a	57.8595 a	66.0444 a	70.6889 a	72.2538 a	15.859
OHGBS	1.0758 a	1.0558 ab	0.8126 bc	0.7596 c	0.8066 bc	0.7476 c	0.6163 c	0.2501
GBN	0.5218 e	2.1840 d	2.8071 bcd	3.8142 ab	3.7410 abc	4.3911 a	2.3367 cd	1.4053
**Indole**	3.1435 a	3.4327 a	2.3546 b	2.2433 b	2.4553 b	1.8550 b	1.9391 b	0.6758
GBS	0.8221 ab	0.9058 a	0.5519 c	0.8544 ab	0.6670 bc	0.4487 c	0.5871 c	0.2211
MeOHGBS	0.8138 a	0.7499 a	0.5995 ab	0.2961 b	0.6999 a	0.3434 b	0.3574 b	0.317
NeoGBS	0.4317 b	0.7212 a	0.3907 b	0.3332 b	0.2818 b	0.3152 b	0.3783 b	0.1847
**Aromatic**								
GNT	0 c	0 c	1.1196 b	2.3373 a	2.0415 ab	1.8546 ab	1.6305 ab	1.079

GSL	Seeds							
	**Br163 (LGBN) C6**	**Br163 (LGBN) C3**	**Br163 (LGBN) C1**	**Br163-C0**	**Br163 (HGBN) C1**	**Br163 (HGBN) C3**	**Br163 (HGBN) C6**	**LSD**
**TOTAL**	26.3107 e	45.4910 d	57.4260 c	64.2002 bc	76.6739 a	76.4832 a	70.9218 ab	7.3488
**Aliphatic**	24.4785 e	43.7115 d	55.5616 c	61.1274 bc	73.5434 a	73.2648 a	67.7847 ab	7.0294
PRO	0.2229 c	0.5515 bc	1.4031 a	0.9499 ab	0.8980 b	0.9991 ab	0.3818 c	0.4632
GAL	-	-	-	-	-	-	-	-
GNL	0.03592 a	0.01624 a	0 a	0.02034 a	0 a	0 a	0 a	0.0363
GNA[1]	23.6534 d	42.4282 c	53.3675 b	59.0086 b	71.4833 a	71.1349 a	66.5211 a	6.8445
OHGBS	1.6744 b	1.6273 b	1.7693 b	2.7032 a	2.9778 a	2.6972 a	2.5762 a	0.5039
GBN	0.5662 d	0.7154 c	0.7911 bc	1.1485 a	1.1620 a	1.1308 a	0.8818 b	0.091
**Indole**	1.8322 b	1.7499 b	1.8167 b	3.0728 a	3.1305 a	3.0482 a	2.9155 a	0.5236
GBS	0.1579 c	0.07601 d	0.04741 d	0.2597 b	0.1418 c	0.3030 ab	0.3241 a	0.0475
MeOHGBS	0 b	0.01124 b	0 b	0.1038 a	0.01097 b	0.03181 b	0.01518 b	0.0446
NeoGBS	0 b	0.03537 a	0 b	0.006125 b	0 b	0.01620 b	0 b	0.017
**Aromatic**								
GNT	0 c	0.02962 bc	0.04765 bc	0 c	0 c	0.1702 ab	0.2216 a	0.1417
